# Women’s educational attainment, full-time farming, and household dietary quality in rural China

**DOI:** 10.3389/fnut.2025.1560455

**Published:** 2025-06-02

**Authors:** Rong Li, Linxiang Ye, Jingwei Han

**Affiliations:** ^1^Institute of Food and Strategic Reverse, Nanjing University of Finance and Economics, Nanjing, China; ^2^School of Economics and Management, Huizhou University, Huizhou, China; ^3^College of Economics, Nanjing University of Finance and Economics, Nanjing, China

**Keywords:** women’s educational attainment, dietary quality, full-time farming, diet balance index, China food pagoda

## Abstract

Diet serves as the primary source of nutrition, and dietary quality determines individuals’ nutritional status. Poor dietary quality may result in malnutrition such as undernutrition and overnutrition. This study examines the association between women’s educational attainment and rural household dietary quality and the heterogeneity in this association when accounting for the employment status of male and female household heads. We use dietary intake data for 2,069 households from the first round of China Rural Revitalization Survey (2020) and conceptualize dietary quality using the Diet Balance Index (DBI). Multiple linear regression indicates that a one-standard-deviation increase in women’s years of education reduces dietary imbalance by 3.65%, overconsumption by 6.51%, and underconsumption by 2.09%. Specifically, higher education attainment is associated with less inadequate consumption of milk and fish and more balanced meat intake in rural households. Heterogeneity analysis shows that the positive effect of women’s education on dietary quality is stronger when women engage in on-farm employment, while it is more pronounced in households where men are employed in off-farm work. The findings of this research provide theoretical support for improving the nutritional status of rural residents in China and other developing regions by allocating more educational resources and enhancing access to education for rural women.

## 1 Introduction

Over the past 4 decades since major economic and social reforms, the dietary quality of rural residents in China has continuously improved (1, 2). However, rural residents still face the dilemma of low dietary quality stemming from an irrational dietary structure. First, rural residents have insufficient dietary diversity. Data from 2,069 households in the 2020 China Rural Revitalization Survey (CRRS) show that a large proportion of rural households did not consume milk (60%), beef and mutton (73%), fish (49%), soybeans (48%), and chicken (34%). Second, compared with urban residents, there are still significant urban–rural differences in dietary quality. Rural residents have higher levels of both insufficient and excessive dietary intake. For example, their intake of vegetables, fish and shrimp, milk, and soybeans remains low, while cereal intake is excessive (3). Third, compared with the recommended dietary pattern outlined in the Chinese Dietary Guidelines 2016 (CDG 2016) (a guideline for a healthy diet released by the National Health Commission of China), most rural residents in China consume more animal meat and less aquatic products and milk. For example, in 2020, the per capita intake of livestock and poultry meat among rural residents was 124 g/day, exceeding the recommended amount of 40–75 g/day, whereas the per capita milk intake was 23 g/day, falling below the recommended 300–500 g/day. Overall, the diversity, moderation, and balance of dietary quality among rural residents in China are inadequate.

Dietary quality, a key determinant of individual nutritional intake, is directly related to their nutritional and health status. Poor dietary quality increases the likelihood of malnutrition—including micronutrient deficiencies and overnutrition associated with overweight and obesity—thereby posing risks to health and wellbeing. For instance, low dietary quality increases the risk of dietary-related chronic diseases (4, 5). Thus, implementing effective measures to improve dietary quality is not only critical for enhancing the nutritional status of rural residents but also contributes to reducing household health maintenance costs in rural China.

Studies in the literature have found that dietary quality in China and other developing countries is directly linked to increases in household income (6, 7), food consumption habits of rural residents (8, 9), agricultural production diversification and rural food market development (10–12), as well as government intervention policies such as government transfer payment policies (13). Among these, education is one factor that appears to have a fundamental influence on dietary quality of households (14–20). Because members of higher educational attainment tend to know more about food nutrition (21, 22), they are also more inclined to healthy dietary habits (23).

Other studies have examined the role of information processing channels (24), the adoption of health-promoting behaviors (25), and perceived control (17) in the relationship between educational attainment and dietary quality. People of higher educational attainment would adopt healthy behaviors such as conscious control of salt, sugar, and oil incorporation to improve dietary quality. In addition to income and the price of goods, consumer decisions are influenced by social class. Social epidemiologists have argued that education levels enable people to rise up the social class hierarchy, which in turn gives them more conviction and a higher degree of self-control in their lives. This feeling of control is reflected in the higher quality of their diets.

Nevertheless, these studies have not adequately addressed whether educational attainment helps narrow the gap between actual dietary pattern and recommended balanced diets in China Food Pagoda 2016 (CFP 2016). Different food varies in nutrient composition and beneficial components. Insufficient intake of specific food groups may affect health by reducing the intake of essential micronutrients, whereas excessive consumption often leads to caloric surpluses, increasing the risk of health issues such as obesity and cardiovascular diseases. Therefore, only a balanced diet can optimally meet the body’s energy and nutrient requirements. Thus, to accurately reflect the nutritional and health status of rural Chinese residents, this study employs the Chinese Dietary Balance Index (DBI_16) to comprehensively evaluate the dietary quality.

On the other hand, the moderating effect of employment status (i.e., non-farm employment vs. on-farm employment) on the relationship between educational attainment and dietary quality has been rarely discussed. Within traditional household labor division framework, women mainly play the role of caring for family members in rural China. They tend to take more responsibility for food selection and cooking compared to men. However, when rural women engage in non-farm work, the time available for household care tends to decrease. Therefore, it is essential to account for women’s employment status when examining the relationship between educational attainment and dietary quality.

In this study, we use dietary intake data for 2,069 households from the first round of China Rural Revitalization Survey (2020) and measure dietary quality using the Diet Balance Index (DBI), to estimate the association between women’s educational attainment and dietary quality. The study has three goals: first, to apply the adjusted Chinese Dietary Balance Index-16 (DBI_16) to assess the dietary quality of rural households in China in 2020; second, to examine whether an increase in women’s educational attainment contributes to the enhancement of dietary quality; third, to estimate the heterogeneous impact of women’s educational attainment on dietary quality among women and men with diverse employment statuses within the same household. It is meaningful to discuss the relationship between women’s educational attainment and household dietary quality. Unlike other established demographic traits, education is more influenced by later policy intervention.

## 2 Data and methods

### 2.1 Study setting: the rural China context

China’s agricultural production is undergoing a transformation from traditional fragmented operations toward large-scale, specialized production systems. The number of specialized rice/wheat farms and commercial fruit–vegetable cooperatives has grown rapidly. According to the Ministry of Agriculture and Rural Affairs of China, the number of farmers engaged in large-scale operations (i.e., individual operation areas exceeding 8 acres) exceeds 3 million, with their total cultivated land area surpassing 66 million acres in 2022.

By 2023, the proportion of large-scale pig farms (≥500 head) reached 68%, commercial layer and broiler farms (≥20,000 birds) accounted for over 80%, and large-scale dairy farms (≥100 cows) represented 76% of total operations.

At the same time, with the advancement of marketization, rural residents in China have largely overcome the constraints of agricultural production on food consumption, gradually shifting from a self-sufficiency-based consumption pattern to a market-oriented pattern. Data from CRRS (2020) show that only 20% of rural households consume self-grown rice, with 80% relying on market purchases; 13% consume self-grown wheat, while the remaining 87% obtain wheat exclusively through markets. The commercialization rate is notably higher for meat products, with 91% of pork consumption sourced from markets, compared to 55% for vegetables. Notably, 45% of rural households still maintain the traditional practice of cultivating vegetables in their backyard for self-consumption.

The dietary pattern in most regions of China is dominated by plant food, supplemented by animal products. Staple food remains the primary source of dietary energy for rural residents in China, but the consumption has been declining annually. As income increases, rural residents tend to consume more animal products, particularly meat.

In general, with the transformation of China’s agriculture from self-sufficiency to marketization and the continuous improvement of rural residents’ income, the food sources of rural residents have become increasingly dependent on the market. The development of the rural food market has enriched rural residents’ food consumption choices and improved their dietary diversity. However, it is weakly associated with dietary quality, i.e., nutrient adequacy. One reason is that the consumption of different food groups may deviate from the recommended dietary pattern in CFP 2016. When such deviations are significant, dietary diversity may weaken the potential association with micronutrient adequacy. In other words, “food accessibility” does not necessarily equate to “buying appropriately.”

### 2.2 Research hypothesis

“Food accessibility” does not necessarily equate to “buying appropriately.” Conversely, education serves to enhance dietary quality by improving cognitive capabilities and income levels. Previous studies have demonstrated that individuals with higher educational attainment are more likely to acquire knowledge about food nutrition (21, 22) and are more prone to adopting healthy eating habits (13). Education empowers individuals to ascend the social class hierarchy, which in turn promotes greater self-confidence and self-control in life (16). For instance, women with higher education exhibit a higher dietary quality compared to those with lower education levels due to their ability to regulate food choices for themselves and their families (17).

In traditional Chinese families, women typically take on the primary responsibility for food selection and preparation, acting as the crucial decision-makers in household food procurement and cooking. Consequently, women have a more direct impact on household dietary quality. Based on the empirical evidence presented above, this study proposes the following hypothesis:

*H1*: An increase in women’s educational attainment contributes to the improvement of rural household dietary quality.

The correlation between women’s educational attainment and dietary quality varies by the employment statuses of male and female household heads. Notably, within households where men engage in off-farm work, the impact of women’s educational attainment on household dietary quality is more significant. This may be attributed to the fact that men’s off-farm or part-time employment increases off-farm income, thereby expanding families’ available food choices (26). In addition, men working away from home enhance women’s empowerment (27), endowing them greater decision-making authority over household food consumption (28–30).

Conversely, within households where women engage in on-farm work, the influence of women’s educational attainment on household dietary balance is more pronounced. This may be attributed to the fact that rural women, as primary caregivers, engaging in off-farm or part-time farming employment away from home will reduce the time available for family care (31). Under such circumstances, female heads of household who work away from home find it difficult to improve household dietary quality (32–34). It is evident that women engaged in on-farm work strengthen the impact of women’s educational attainment on household dietary quality. Therefore, this study proposes the following hypothesis:

*H2*: The positive effect of women’s education on dietary quality is more pronounced when women engage in on-farm employment, whereas this effect is stronger in households where men are employed in off-farm work.

### 2.3 CRRS dataset

The China Rural Revitalization Survey (CRRS) is an ongoing national survey on agriculture, rural areas, and rural residents, implemented by the Rural Development Institute Chinese Academy of Social Sciences. The survey was conducted every 2 years starting from 2020. The data cover 300 villages and 3,833 rural households across 10 provinces of China (i.e., Guangdong, Zhejiang, Shandong, Anhui, Henan, Heilongjiang, Guizhou, Sichuan, Shaanxi, and Ningxia). These provinces include the eastern, central, western, and northeastern regions, and the well-developed region and the poor region. The currently released data are from the 2020 survey round.

The survey provides sufficient data for our study. We focus on the total amount of food consumed by the permanent household population through all channels (i.e., purchasing, granting, and self-planting), as well as data related to the basic information of household members (gender, age, educational attainment and employment status, etc.), net income of the rural household, health behaviors, and the basic conditions of the village.

Since family members include the elderly, children and adults, people of different ages, genders and activity levels have different food requirements. It is therefore customary in international studies to express the number of persons in a population in terms of its equivalent in adult males (or “consumption units”) (35). Many different systems are used in effecting this conversion. In this study, an adult man engaged in light physical activity (whose energy requirement is usually set as a standard value, e.g., 2,250 kcal/day) is taken as the reference standard, and the food consumption of other family members is converted into the consumption equivalent to that of this standard adult man, and this converted amount is called adult equivalent (36). Then, dividing the total household consumption by the number of adult equivalent is called the per capita food consumption of each family.

In this study, samples with daily caloric intake below 800 kcal or above 10,000 kcal were excluded, along with those containing missing data. Following these exclusions, data from 2,069 rural households were retained for analysis.

### 2.4 Empirical model

The impact of women’s educational attainment on dietary quality can be modeled as follows:


$${\mathrm{DBI}}_{i}=\beta_{0}+\beta_{1}\mathrm{Wo}\_{\mathrm{edu}}_{i}+\varphi_{i}\sum {\text{controls}}_{i}+\mu_{i}$$

where ${\mathrm{DBI}}_{i}$ refers to dietary balance index indices of household i in year 2020, including three indicators of diet quality distance (DQD), high bound score (HBS), and low bound score (LBS); $\mathrm{Wo}\_{\mathrm{edu}}_{i}$ refers to educational attainment of women head of household i; ${\text{controls}}_{i}$ refers to all other variables, including individual and household characteristics (e.g., women’s occupation, men’s occupation, per capita net income, household size, and ratio of elderly and children) and village characteristics (level of village distribution, distance between the village committee and the county government, and the terrain of the village).

In addition, we investigate the heterogeneous effects of women’s education attainment on household dietary quality across subgroups: households where female heads engage in on-farm work at home, and households where male heads are employed in off-farm work.

### 2.5 Measurement of key variables

#### 2.5.1 Dietary quality

We employed the Revised Dietary Balance Index-16 [DBI_16, (3)] to evaluate dietary quality by quantifying the deviation between residents’ dietary patterns and the recommended patterns according to Chinese Dietary Guidelines 2016 (CDG 2016) and China Food Pagoda 2016 (CFP 2016). The DBI_16 indicator evaluates the balance degree of diet through the consumption of foods such as cereals (grains, potatoes, and beans), vegetables, meat (pork, chicken, beef, and mutton), eggs, dairy, and fish. Specifically, each food item receives score “0” if the real consumption is within the recommended consumption interval, and it shows that the intake of this food group is reasonable. The sore is positive if the real consumption is higher than the recommended consumption, and the score increased “1” when real consumption increased a certain number of grams. The sore is negative if the real consumption is lower than the recommended consumption, and the score decreased “1” when real consumption decreased a certain number of grams. Then, the positive scores are summed up as the High Bound Score (HBS), which reflect the degree of dietary overconsumption. The absolute values of negative scores are summed up as the Low Bound Score (LBS), which reflected the degree of dietary underconsumption. Finally, the positive scores and the absolute values of negative scores of six food groups are summed up as Diet Quality Distance (DQD), with a score of “0” indicating a balanced diet and a higher score indicating a greater degree of dietary imbalance.

#### 2.5.2 Women’s educational attainment

The primary explanatory variable of women’s educational attainment is measured by years of schooling completed. 0 represents no schooling. 6 represents graduation from primary school. 9 represents graduation from junior high school. 12 indicates graduation from high school, secondary school, and vocational school. 15 indicates graduation from junior college. 16 represents the education level is undergraduate degree. In the sample, female household heads have an average of 6 years of schooling (primary school level) and a maximum of 16 years (undergraduate degree).

#### 2.5.3 Employment status of men and women

The employment status of men and women is measured as three categories: full-time farming, off-farm employment, and concurrent employment (concurrent employment refers to farmers returning to their rural hometowns for agricultural production during busy farming seasons and seeking non-agricultural employment during slack agricultural periods). We assign a value of 1 to denote off-farm employment or concurrent employment and 0 for full-time farming.

#### 2.5.4 Other covariates

We measure the healthy eating behaviors with the questions of the questionnaire: “whether consciously learning health or health knowledge,” “whether consciously controlling sugar intake,” “whether consciously controlling salt intake,” “whether consciously controlling the intake of edible oil,” and “whether consuming health products.” The score for health behavior is the sum of the number of the above health behaviors. If there is one health behavior, the score is 1, and so on.

The variable of information access via online platforms is scored according to the question in the survey questionnaire: “If you have daily needs, can you obtain relevant information through your mobile phone or network at any time by yourself?” If the answer is “Completely,” the score is 1; if it is “Sometimes,” the score is 0.5; and if it is “Rather difficult,” the score is 0.

The per capita net income of rural households is the net income of rural household divided by the total household size in 2019. The income includes planting, livestock, forestry and fruit farming, fishing, non-agricultural operating, wage, property, and transfer. We also control individual characteristics, household characteristics, and village characteristics. We also control the provincial dummy variables. Table 1 provides a brief summary and definition of all variables.

**Table 1:** Descriptive statistics and variable definitions (*N* = 2,067 households; Data source: CRRS 2020).

Variables	Mean	St.dev.	Min	Max	Definition
DQD	17.42	5.66	0	35	Diet quality distance
HBS	6.14	5.65	0	20	High bound score
LBS	11.27	3.89	0	32	Low bound score
Wo_edu	7.40	3.51	0	16	Years of schooling for women
Health behavior	2.45	1.72	0	5	Health behavior score
Dig_skill	0.64	0.41	0	1	Obtaining information through the Internet: 1 = complete, 0.5 = sometimes, 0 = difficult
Income	26,364	3,357	14,580	300,888	Per capita household income in 2019
Wo_career	0.34	0.47	0	1	1 = off-farm employment and concurrent employment, 0 = full-time farming
Man_career	0.52	0.50	0	1	1 = off-farm employment and concurrent employment, 0 = full-time farming
Hhsize	3.59	1.61	1	14	Number of household members
Old_ratio	0.21	0.30	0	1	People aged 60 and over ratio of household members
Child_ratio	0.12	0.18	0	2	Children aged 3–12 ratio of household members
Distribution	0.60	0.35	0	1	1 = express delivery to households, 0 = express delivery is not available to households
Distance	22.70	17.13	1	125	Distance between village committee and county government (km)
Terrain	0.65	0.48	0	1	1 = below 500 meters in altitude, 0 = above 500 meters in altitude

Concurrent employment refers to farmers returning to their rural hometowns for agricultural production during busy farming seasons and seeking non-agricultural employment during slack agricultural periods.

## 3 Results

### 3.1 Dietary quality of Chinese rural households

**Figure 1:**
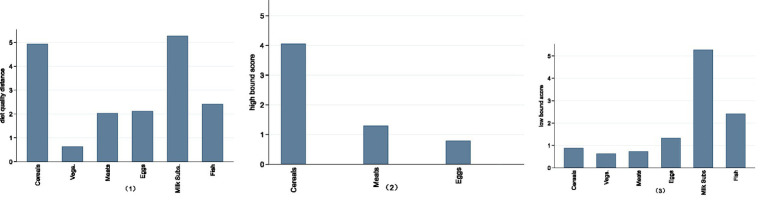
DBI_16 scores of different food groups of Chinese rural households in 2020. (1) The horizontal axis represents different food groups, and the vertical axis represents the DBI_16 score. The Diet Quality Distance comprehensively reflects issues in dietary patterns. The High Bound Score indicates the degree of dietary overconsumption, while the Low Bound Score reflects the degree of dietary underconsumption. All three indicators are derived from China Food Pagoda (CFP 2016). A higher score signifies a greater degree of dietary imbalance and lower dietary quality. (2) In this figure (2), there are no high bound scores for vegetables, dairy products and fish.

We first present the DBI_16 scores of different food groups of Chinese rural households in 2020, which reflected the balance degree of various food intakes. The results are presented in Figure 1. Our data reveal that Chinese rural households have an overconsumption of cereals and meats and a severe deficient intake of dairy products and fish. Overall, cereals, dairy products, and fish present a moderate dietary imbalance, while meat and eggs exhibit a low-level dietary imbalance. The intake of vegetables is relatively appropriate.

### 3.2 Impact of women’s educational attainment on the dietary quality of Chinese rural households

Table 2 presents the test results regarding the impact of women’s educational attainment on the dietary quality of households. With the inclusion of all controls in the model, we find that a one-standard-deviation increase in women’s years of education reduces dietary imbalance by 3.65%, overconsumption by 6.51%, and underconsumption by 2.09%. The research outcomes indicate that women’s educational attainment promotes the dietary quality of rural households in China.

**Table 2:** Impact of women’s educational attainment on dietary quality.

Explanatory variables	Dietary quality		
	Diet quality distance	High bound score	Low bound score
Wo_edu	−0.181^***^	−0.114^**^	−0.067^**^
	(−4.82)	(−3.07)	(−2.64)
Wo_career	0.074	0.008	0.082
	(0.25)	(0.03)	(0.42)
Man_career	−0.601^*^	−0.551^*^	−0.050
	(−2.24)	(−2.08)	(−0.27)
lnIncome	−0.496^***^	0.239	−0.735^***^
	(−3.94)	(1.93)	(−8.63)
Hhsize	−0.280^**^	−0.652^***^	−0.373^***^
	(−3.17)	(−7.50)	(−6.24)
Old_ratio	1.039^*^	2.176^***^	1.137^***^
	(2.47)	(5.24)	(3.99)
Child_ratio	−0.739	−0.233	−0.972
	(−0.95)	(−0.30)	(−1.85)
Distribution	−0.018	−0.256	0.238
	(−0.05)	(−0.73)	(0.99)
Distance	0.030^***^	0.025^***^	0.005
	(3.93)	(3.34)	(0.93)
Terrain	−0.237	−0.208	−0.445^*^
	(−0.86)	(−0.76)	(−2.37)
Constant	24.260^***^	6.273^***^	17.980^***^
	(18.10)	(4.75)	(19.81)
*N*	2,069	2,069	2,069
Adjusted *R*-squared	0.058	0.078	0.086

(1) *, **, and *** indicate significance at 10, 5, and 1% levels, respectively.

(2) Models estimated using multiple linear regression, and value in brackets is t statistics.

(3) DQD, Diet Quality Distance; HBS, High Bound Score; LBS, Low Bound Score.

We further estimate the impact of women’s educational attainment on the DBI for each food group separately, with the results presented in Table 3. We observe that women with a higher level of education tend to reduce cereal consumption, and the degree of insufficient intake increases. This suggests that better-educated women prioritize family members’ weight management and are concerned that consuming staple foods such as rice and noodles may contribute to obesity. Conversely, highly educated women are more likely to choose a more diverse set of foods, incorporating more meat, dairy products, and fish, thereby decreasing reliance on traditional staple foods.

**Table 3:** Impact of women’s educational attainment on the dietary quality of each food group separately.

Explanatory variables	Dietary quality of each food group							
	LBS of cereals(1)	HBS of cereals(2)	LBS of vegetables(3)	LBS of meat(4)	HBS of meat(5)	LBS of eggs(6)	LBS of milk(7)	LBS of fish(8)
Wo_edu	0.056^***^	−0.169^***^	0.005	−0.023^**^	−0.044^***^	−0.029^*^	−0.037^***^	−0.041^***^
	(4.87)	(−5.21)	(0.98)	(−2.66)	(−4.00)	(−2.43)	(−4.95)	(−3.72)
Wo_career	0.206^*^	−0.020	0.043	−0.114	0.097	0.054	0.041	−0.312^***^
	(2.32)	(−0.08)	(1.03)	(−1.75)	(1.15)	(0.60)	(0.72)	(−3.67)
Man_career	0.208^*^	−0.299	−0.062	−0.032	−0.139	−0.005	−0.110^*^	−0.050
	(2.54)	(−1.29)	(−1.58)	(−0.53)	(−1.78)	(−0.06)	(−2.10)	(−0.63)
lnIncome	0.039	−0.139	0.020	−0.292^***^	0.340^***^	−0.072	−0.158^***^	−0.271^***^
	(1.01)	(−1.28)	(1.09)	(−10.34)	(9.32)	(−1.84)	(−6.43)	(−7.34)
Hhsize	0.159^***^	−0.428^***^	0.092^***^	−0.014	−0.082^**^	0.134^***^	0.002	−0.001
	(5.91)	(−5.63)	(7.16)	(−0.69)	(−3.19)	(4.87)	(0.09)	(−0.00)
Old_ratio	−0.301^*^	1.730^***^	−0.039	−0.052	0.162	−0.417^**^	−0.153	−0.176
	(−2.34)	(4.77)	(−0.63)	(−0.55)	(1.32)	(−3.18)	(−1.85)	(−1.43)
Child_ratio	0.324	−0.284	−0.137	−0.158	0.157	−0.314	−0.822^***^	0.135
	(1.36)	(−0.42)	(−1.21)	(−0.90)	(0.70)	(−1.30)	(−5.39)	(0.59)
Distribution	0.172	−0.272	−0.017	−0.035	−0.012	0.059	−0.126	0.185
	(1.59)	(−0.89)	(−0.34)	(−0.44)	(−0.12)	(0.54)	(−1.82)	(1.78)
Distance	−0.005^*^	0.017^*^	0.001	−0.003	0.01^***^	0.01^***^	0.01	0.01
	(−2.20)	(2.56)	(0.65)	(−1.94)	(4.47)	(3.75)	(1.42)	(0.67)
Terrain	0.172^*^	0.041	−0.046	0.232^***^	−0.081	−0.411^***^	0.010	−0.403^***^
	(2.03)	(0.17)	(−1.14)	(3.73)	(−1.00)	(−4.76)	(0.19)	(−4.95)
Constant	−0.736	7.791^***^	0.138	3.810^***^	−2.207^***^	1.891^***^	7.267^***^	5.612^***^
	(−1.80)	(6.74)	(0.71)	(12.65)	(−5.67)	(4.53)	(27.65)	(14.25)
*N*	2,069	2,069	2,069	2,069	2,069	2,069	2,069	2,069
Adjusted *R*-squared	0.081	0.067	0.028	0.068	0.073	0.051	0.058	0.072

(1) *, **, and *** indicate significance at 10, 5, and 1% levels, respectively.

(2) Models estimated using multiple linear regression, and value in brackets is t statistics.

(3) There are no high bound scores for vegetables, dairy products, and fish.

(4) The HBS for eggs is not significant because space constraints are not listed.

However, improvement in women’s educational attainment can effectively reduce the severity of insufficient intake of dairy products and fish in rural households. Specifically, a one-standard-deviation increase in women’s educational attainment is associated with a 2.46% decrease in dairy insufficiency and a 5.96% decrease in fish insufficiency, relative to the sample mean. Regarding balanced meat consumption, the same increase in educational attainment leads to a 10.98% reduction in under-consumption and an 11.92% reduction in over-consumption of meat.

### 3.3 Heterogeneous effects

We further conduct grouped regression analyses based on the employment status of male and female household heads, aiming to examine the heterogeneous effects of women’s educational attainment on dietary quality.

#### 3.3.1 By the employment status of men

First, we divide the total sample into two subgroups based on male household heads’ employment status: off-farm employment (including part-time farming) and full-time farming. The results are presented in Table 4. We find that the effect of women’s educational attainment on dietary quality is more significant in households where male heads engage in off-farm work.

**Table 4:** Heterogeneous impact of women’s educational attainment on dietary quality at different employment statuses of men.

Explanatory variables	Dietary quality					
	Diet quality distance		High bound score		Low bound score	
	Off-farm (1)	On-farm (2)	Off-farm (3)	On-farm (4)	Off-farm (5)	On-farm (6)
Wo_edu	−0.179^***^	−0.178^**^	−0.121^*^	−0.100	−0.078^*^	−0.058
	(−3.50)	(−3.17)	(−2.43)	(−1.78)	(−2.15)	(−1.60)
Wo_career	−0.246	0.276	−0.077	0.178	−0.169	0.098
	(−0.72)	(0.51)	(−0.23)	(0.33)	(−0.70)	(0.28)
lnIncome	−0.616^***^	−0.390^*^	0.211	0.248	−0.827^***^	−0.638^***^
	(−3.57)	(−2.11)	(1.25)	(1.35)	(−6.78)	(−5.33)
Hhsize	−0.352^**^	−0.233	−0.679^***^	−0.650^***^	0.327^***^	0.417^***^
	(−2.82)	(−1.83)	(−5.58)	(−5.13)	(3.72)	(5.05)
Old_ratio	0.994	1.185^*^	2.417^***^	2.080^***^	−1.423^**^	−0.896^*^
	(1.55)	(2.08)	(3.85)	(3.67)	(−3.14)	(−2.43)
Child_ratio	−1.099	−0.222	−0.113	0.806	−0.986	−1.027
	(−1.11)	(−0.18)	(−0.12)	(0.65)	(−1.41)	(−1.27)
Distribution	0.068	−0.076	−0.175	−0.341	0.242	0.266
	(0.14)	(−0.14)	(−0.38)	(−0.65)	(0.72)	(0.77)
Distance	0.024^*^	0.036^**^	0.020	0.030^**^	0.004	0.006
	(2.20)	(3.28)	(1.88)	(2.76)	(0.51)	(0.82)
Terrain	0.035	−0.563	0.424	−0.056	−0.389	−0.507
	(0.09)	(−1.34)	(1.18)	(−0.13)	(−1.50)	(−1.86)
Constant	25.13^***^	23.03^***^	6.123^***^	6.123^**^	19.01^***^	16.90^***^
	(13.34)	(11.81)	(3.33)	(3.15)	(14.28)	(13.37)
*N*	1,079	990	1,079	990	1,079	990
Adjusted *R*-squared	0.045	0.045	0.070	0.067	0.079	0.086
Empirical *p*-value	0.074*		0.092*		0.042*	

(1) *, **, and *** indicate significance at 10, 5, and 1% levels, respectively.

(2) Models estimated using multiple linear regression, and value in brackets is t statistics.

(3) Empirical *p*-value is used to test the significance of the differences in the wo_edu coefficient between groups, which is obtained by bootstrapping 1,000 times.

#### 3.3.2 By the employment status of women

We further divide the total sample into two subgroups based on female household heads’ employment status: off-farm employment (including part-time farming) and full-time farming. The results are presented in Table 5. We find that the influence of women’s educational attainment on household dietary balance is more pronounced among households where women engage in full-time farming.

**Table 5:** Heterogeneous impact of women’s educational attainment on dietary quality at different employment statuses of women.

Explanatory variables	Dietary quality					
	Diet quality distance		High bound score		Low bound score	
	Off-farm (1)	On-farm (2)	Off-farm (3)	On-farm (4)	Off-farm (5)	On-farm (6)
Wo_edu	−0.164^*^	−0.189^***^	−0.085	−0.129^**^	−0.080	−0.059^*^
	(−2.54)	(−4.04)	(−1.28)	(−2.85)	(−1.66)	(−1.96)
Man_career	−0.936	−0.438	−0.696	−0.474	−0.240	0.037
	(−1.88)	(−1.36)	(−1.37)	(−1.52)	(−0.65)	(0.18)
lnIncome	−0.550^**^	−0.487^**^	0.284	0.198	−0.834^***^	−0.686^***^
	(−2.59)	(−3.11)	(1.31)	(1.30)	(−5.27)	(−6.79)
Hhsize	−0.403^**^	−0.228^*^	−0.781^***^	−0.608^***^	0.378^**^	0.379^***^
	(−2.61)	(−2.11)	(−4.96)	(−5.77)	(3.29)	(5.42)
Old_ratio	1.138	1.003^*^	3.052^***^	1.877^***^	−1.914^**^	−0.875^**^
	(1.41)	(2.00)	(3.69)	(3.85)	(−3.17)	(−2.70)
Child_ratio	1.590	−1.873	1.794	−0.513	−0.204	−1.360^*^
	(1.22)	(−1.93)	(1.34)	(−0.55)	(−0.21)	(−2.18)
Distribution	0.416	−0.168	−0.164	−0.267	0.580	0.099
	(0.69)	(−0.39)	(−0.26)	(−0.63)	(1.28)	(0.35)
Distance	0.034^*^	0.028^**^	0.032^*^	0.021^*^	0.001	0.007
	(2.55)	(2.98)	(2.42)	(2.29)	(0.11)	(1.17)
Terrain	0.114	−0.419	0.473	0.061	−0.359	−0.479^*^
	(0.26)	(−1.17)	(1.06)	(0.17)	(−1.10)	(−2.08)
Constant	24.43^***^	24.38^***^	5.476^*^	6.942^***^	18.95^***^	17.44^***^
	(10.60)	(14.55)	(2.33)	(4.26)	(11.02)	(16.13)
*N*	703	1,366	703	1,366	703	1,366
Adjusted *R*-squared	0.041	0.053	0.073	0.071	0.089	0.082
Empirical *p*-value	0.012*		0.064*		0.068*	

(1) *, **, and *** indicate significance at 10, 5, and 1% levels, respectively.

(2) Models estimated using multiple linear regression, and value in brackets is t statistics.

(3) Empirical *p*-value is used to test the significance of the differences in the wo_edu coefficient between groups, which is obtained by bootstrapping 1,000 times.

### 3.4 Robustness check

#### 3.4.1 Instrumental variable method

The regression results mentioned above might suffer from endogeneity issues due to omitted variables. In light of this, we proceed to introduce instrumental variables based on the benchmark model. By leveraging the two-stage least squares (2SLS) methodology, we aim to address the potential endogeneity issues and enhance the robustness and reliability of our analytical findings.

Drawing on previous research, we choose to use the average educational attainment of women in other townships within the same county as an instrumental variable. Table 6 reports the estimation results. The findings show that the educational attainment of women still significantly improves the dietary quality of rural households even after instrumental variables are used to address the potential endogeneity biases. In comparison with the OLS regression results presented in Table 2, we find that the estimated magnitude of the impact of women’s educational attainment on the household dietary quality increases when the instrumental variable method is employed. This suggests that overlooking endogeneity concerns would lead to an underestimation of the impact. Nevertheless, it should be noted that the results obtained from the OLS test still have a certain degree of validity within the overall analysis.

**Table 6:** Estimated results using the instrumental variable approach.

Explanatory variables	Dietary quality		
	Diet quality distance	High bound score	Low bound score
Wo_edu	−0.631^***^	−0.235^*^	−0.396^***^
	(−4.18)	(−2.32)	(−3.91)
Wo_career	0.713	0.220	0.493
	(1.86)	(0.60)	(1.84)
Man_career	−0.522	−0.530^*^	0.008
	(−1.88)	(−2.01)	(0.04)
lnIncome	−0.313^*^	0.288^*^	−0.601^***^
	(−2.21)	(2.17)	(−6.39)
Hhsize	−0.197^*^	−0.630^***^	0.433^***^
	(−2.20)	(−7.30)	(6.83)
Old_ratio	0.436	2.013^***^	−1.577^***^
	(0.86)	(4.08)	(−4.87)
Child_ratio	−0.328	0.344	−0.672
	(−0.40)	(0.47)	(−1.17)
Distribution	0.099	−0.225	0.324
	(0.27)	(−0.64)	(1.30)
Distance	0.022^**^	0.023^**^	−0.001
	(2.64)	(2.89)	(−0.20)
Terrain	0.236	0.335	−0.099
	(0.72)	(1.08)	(−0.47)
Constant	25.09^***^	6.497^***^	18.59^***^
	(17.79)	(4.96)	(19.21)
Kleibergen-Paap LM statistic	120.838 (*p* = 0.000)	120.838 (*p* = 0.000)	120.838 (*p* = 0.000)
Kleibergen-Paap Wald F statistic	143.487	143.487	143.487
χ^2^(1) statistic	10.238 (*p* = 0.001)	0.867 (*p* = 0.352)	11.690 (0.001)
Durbin–Wu–Hausman test	10.67 (*p* = 0.001)	11.12 (*p* = 0.001)	11.84 (*p* = 0.001)

(1) *, **, and *** indicate significance at 10, 5, and 1% levels, respectively.

(2) Value in brackets is t statistics.

#### 3.4.2 Quantile regression

We select three quantiles (1/4, 1/2, and 3/4) to assess the robustness of the “mean regression” results in Table 2, which are obtained using the ordinary least squares (OLS) method. The findings, presented in Table 7, suggest that across different quantiles, women’s educational attainment significantly impacts household dietary quality. Moreover, the signs of the quantile regression coefficients exactly match those of the OLS regression results in Table 2. This correspondence indicates that the OLS model estimates in Table 2 are reliable and robust, validating the initial analysis and strengthening the confidence in the observed variable relationships.

**Table 7:** Quantile regression results of the dietary quality.

Explanatory variables	Quantiles of DQD			Quantiles of HBS			Quantiles of LBS		
	0.25	0.5	0.75	0.25	0.5	0.75	0.25	0.5	0.75
Wo_edu	−0.120^**^	−0.253^***^	−0.195^**^	−0.024^*^	−0.207^***^	−0.233^***^	−0.110^**^	−0.060^*^	−0.032
	(−2.69)	(−5.02)	(−3.18)	(−2.35)	(−3.62)	(−3.46)	(−3.23)	(−2.36)	(−0.88)
Wo_career	−0.044	0.075	−0.343	−0.224	−0.304	0.028	−0.100	−0.082	−0.314
	(−0.13)	(0.19)	(−0.73)	(−0.72)	(−0.69)	(0.05)	(−0.38)	(−0.33)	(−1.13)
Man_career	−0.207	−0.447	−0.944^*^	−0.073	−0.426	−0.855	−0.028	−0.073	−0.123
	(−0.65)	(−1.25)	(−2.16)	(−0.25)	(−1.05)	(−1.78)	(−0.11)	(−0.32)	(−0.48)
lnIncome	−0.590^***^	−0.469^**^	−0.660^**^	0.369^**^	0.264	0.081	−0.759^***^	−0.829^***^	−0.847^***^
	(−3.95)	(−2.80)	(−3.22)	(2.73)	(1.38)	(0.36)	(−6.70)	(−7.73)	(−7.05)
Hhsize	0.021	−0.221	−0.500^***^	−0.276^**^	−0.704^***^	−0.902^***^	0.324^***^	0.419^***^	0.366^***^
	(0.20)	(−1.88)	(−3.48)	(−2.91)	(−5.26)	(−5.72)	(4.07)	(5.58)	(4.35)
Old_ratio	1.145^*^	1.532^**^	1.735^*^	1.864^***^	3.502^***^	2.652^***^	−1.235^**^	−1.062^**^	−0.743
	(2.29)	(2.73)	(2.53)	(4.11)	(5.48)	(3.52)	(−3.26)	(−2.96)	(−1.85)
Child_ratio	−1.019	−0.932	−1.176	0.322	0.698	−1.537	−0.757	−0.945	−0.635
	(−1.10)	(−0.90)	(−0.93)	(0.38)	(0.59)	(−1.11)	(−1.08)	(−1.43)	(−0.86)
Distribution	0.280	0.026	0.044	0.059	−0.265	−0.201	0.014	0.003	0.347
	(0.67)	(0.06)	(0.08)	(0.16)	(−0.49)	(−0.32)	(0.04)	(0.01)	(1.03)
Distance	0.0131	0.037^***^	0.033^**^	0.015	0.031^**^	0.044^**^	0.002	0.001	0.002
	(1.44)	(3.66)	(2.62)	(1.82)	(2.66)	(3.18)	(0.28)	(0.10)	(0.22)
Terrain	−0.081	−0.133	−0.102	0.170	0.083	0.530	−0.442	−0.563^*^	−0.489
	(−0.25)	(−0.36)	(−0.23)	(0.57)	(0.20)	(1.07)	(−1.77)	(−2.39)	(−1.85)
Constant	19.48^***^	23.44^***^	30.56^***^	−1.945	5.572^**^	13.21^***^	16.29^***^	18.81^***^	21.17^***^
	(12.24)	(13.10)	(14.00)	(−1.35)	(2.74)	(5.51)	(13.48)	(16.47)	(16.52)
*N*	2,069	2,069	2,069	2,069	2,069	2,069	2,069	2,069	2,069

(1) *, **, and *** indicate significance at 10, 5, and 1% levels, respectively.

(2) Value in brackets is t statistics.

The robustness test results indicate that neither the instrumental variable (IV) approach nor quantile regression alters the conclusion regarding the impact of women’s educational attainment on household dietary quality, thereby confirming the validity of the earlier OLS estimates in Table 2.

## 4 Discussion

This study investigates the impact of women’s educational attainment on the dietary quality of rural households in China, with a specific focus on the moderating effect of different employment statuses of household heads. The CRRS data (2020) are adopted, and the dietary quality is assessed with Dietary Balance Index. In general, the main findings can be summarized as follows:

First, the findings indicate that a one-standard-deviation increase in women’s years of education is associated with a 3.65% reduction in dietary imbalance, a 6.51% decrease in dietary overconsumption, and a 2.09% decline in dietary underconsumption. Specifically, higher educational attainment is linked to less inadequate intake of milk and fish and more balanced meat consumption in rural households. These associations can be attributed to several underlying mechanisms. For example, education empowers women with enhanced cognitive abilities and self-learning skills, enabling them to acquire dietary and nutritional knowledge beyond formal education and interpret nutritional information, such as food ingredient labels and dietary recommendations. In addition, education strengthens women’s capacity to access online information. More educated women can efficiently identify reliable dietary resources, such as government dietary guidelines and online platforms (e.g., dietary guideline apps), and leverage these tools to inform food choices.

Second, heterogeneity analysis reveals that the association between women’s educational attainment and dietary quality varies by the employment status of male and female household heads. This variation can be attributed to three key mechanisms: (1) Off-farm income earned by male migrant workers significantly expands household food budgets, thereby laying an economic foundation for improving dietary quality. (2) When men engage in off-farm work, women often assume primary responsibility for household food procurement. Compared with less-educated women, those with higher education demonstrate distinct advantages in developing science-based procurement strategies, which directly enhance the nutritional value of household diet. (3) Women engaged in on-farm work can prepare daily meals for their families after field labor, ensuring regular dietary supply. By contrast, if they migrate for off-farm employment, the workplace is typically far from home, and they usually return home only once every year. This reduction in time available for attending to household dietary needs can consequently affect dietary quality.

### 4.1 Study limitations

The main limitation of this study is that cross-sectional data do not capture the dynamic interplay between education, occupation, and dietary quality over time. In addition, we do not examine how women’s educational attainment improves child dietary quality as household dietary quality may differ at the individual level. Future research can build on this body of work by analyzing how the education–occupation–dietary quality relationship evolves over time across diverse subnational regions, exploring the pathways through which women’s educational attainment impacts dietary quality using quantitative methods, and examining how maternal education affects children’s dietary quality. Researchers may anticipate the release of the second and third rounds of China Rural Revitalization Survey (CRRS) data, which could provide more nuanced metrics at the individual level.

### 4.2 Policy implications

These findings highlight the critical role of women’s education in promoting healthier dietary patterns among rural residents in developing countries other than China. This study demonstrates that investing in rural women’s education can effectively address the coexistence of undernutrition and overnutrition stemming from poor dietary quality by promoting balanced consumption of food. Therefore, policymakers should prioritize allocating more educational resources and opportunities for rural women. Second, encouraging male household heads to engage in off-farm work not only increases household income but also empowers women to assume greater decision-making authority over dietary choices, leveraging their improved education to prioritize nutritious options. Third, health education should be strengthened to correct misconceptions such as “staple foods equal carbohydrates” or “staple foods cause obesity.” Cereal-based staples, while rich in starch, are also vital sources of micronutrients and dietary fiber. Moderate intake of these foods is essential for maintaining health.

## Data Availability

Publicly available datasets were analyzed in this study. This data can be found here: China Rural Revitalization Survey: http://rdi.cass.cn/dcsj/202306/t20230607_5643271.shtml.
